# Nonlinear Dynamics of Reaction Time and Time Estimation during Repetitive Test

**DOI:** 10.3390/ijerph19031818

**Published:** 2022-02-05

**Authors:** Elena Ioana Iconaru, Manuela Mihaela Ciucurel, Mariana Tudor, Constantin Ciucurel

**Affiliations:** 1 Department of Medical Assistance and Physical Therapy, University of Pitesti, 110040 Pitesti, Romania; mariana.tudor@upit.ro (M.T.); constantin.ciucurel@upit.ro (C.C.); 2 Department of Psychology, Communication Sciences and Social Assistance, University of Pitesti, 110040 Pitesti, Romania; manuela.ciucurel@upit.ro

**Keywords:** reaction time, time estimation, Weber fraction, temporal variability, nonlinear analysis, Poincaré plot

## Abstract

*(1) Background:* In this research, we aimed to investigate a computational model of repetitive reaction time (RT) and virtual reaction time (VRT) testing. *(2) Methods:* The study involved 180 subjects (50 men, 130 women, mean age 31.61 ± 13.56 years). The data were statistically analyzed through the coefficient of variation (CV) and the Poincaré plot indicators. *(3) Results:* We obtained an excellent level of reliability for both sessions of testing and we put into evidence a relationship of association of the RT and VRT with the subjects’ age, which was more pregnant for RT (*p* < 0.05). For both RT and VRT data series, we determined a consistent closer association between CV and the Poincaré plot descriptors SD_1_, SD_2_ (SD—standard deviation), and the area of the fitting ellipse (AFE) (*p* < 0.01). We reported an underestimation of the time interval of 2 s during the VRT session of testing, with an average value of CV of VRT, the equivalent of the Weber fraction, of 15.21 ± 8.82%. *(4) Conclusions:* The present study provides novel evidence that linear and nonlinear analysis of RT and VRT variability during serial testing bring complementary insights to the understanding of complex neurocognitive processes implied in the task execution.

## 1. Introduction

As a complex neurocognitive function, time estimation is fundamental for human beings because it conditions the adaptative behavior in everyday life settings [1]. The process of time estimation has been intensively studied in animals and humans, but the results of this research are often controversial regarding the precision of different tasks and are interpreted in terms of multiple neuropsychological models, depending on the subject’s age, gender, and interval duration [2,3], type of sensorial stimuli applied [4] or contextual factors during testing [5]. Most studies put into evidence the existence of an internal clock, according to the Scalar Timing Theory, which states that the behavior of an animal is a function of the time since a stimulus began [6,7,8]. On the subject of the short time estimation, within a range of seconds, this process involves complex cognitive functions, which depend on multiple brain regions, a special role assigned to the short-term and working memory [9].

The average timing precision and variability measured by the coefficient of variation (CV) are parameters used in time estimation [10], in accordance with the Scalar Timing Theory [6]. CV has been also used to estimate the dynamics of the Weber fraction (WF) in time estimation tasks because the standard deviation of interval estimation is proportional to the absolute time interval, according to Weber’s law or the hypothesis of a scalar property for time [11]. However, some authors consider the WF as inconstant in multiple interval testing or during special conditions of testing (when very short durations, <100 ms, were timed or when timing tasks varied in difficulty) [12,13]. Additionally, a violation of the scalar property between 1 and 1.9 s was put into evidence, with an improvement of accuracy in tests with multiple intervals required [13].

The link between time estimation and reaction time (RT) has been demonstrated over time through numerous experimental research [14]. Thus, intricate neuropsychological mechanisms surround these physiological processes, humans being susceptible to neutrally and temporal predictability in response times [15]. A recent generous research perspective was provided by the application of mathematical models for interpreting the variability of RT in different influential test conditions [16]. A general conclusion of such a study, based on the processing of cortical biosignals, consists of the fact that variations in RT, as a specific characteristic of human behavior, are due to temporal variations of neural activity [17]. In a recent study, the approach of which we want to continue with, we demonstrated the usefulness of applying the Poincaré plot method for the study of nonlinear dynamics of RT during a repetitive computerized visual test [18].

The Poincaré plot is a statistical technique used to analyze, from a geometrical point of view, the correlation between two consecutive data points in a time-series, with large applications in the study of fluctuations in physiological rhythms of biological systems [19,20]. The resulting plot is an ellipse that puts into evidence the nonlinear dynamics of the investigated variable, based on four descriptors: SD_1_, SD_2_, the area of the fitting ellipse (AFE), and the fraction between SD_1_ and SD_2_ (SD_1_/SD_2_). Practically, SD_1_ represents the small axis of the geometrical elliptical representation of a time series of data and indicates their variation on a short time scale, while SD_2_ is the large axis of the ellipse and shows the data variation on a long-term scale [19,20,21]. The SD_1_/SD_2_ parameter, as the relative balance between SD_1_ and SD_2_, reflects the clarity and linearity of the scatter pattern, with regards to the ratio between short- and long-term variabilities of the time series of data, associated with the degree of the system’s physiological disorder depth [22,23]. The resulted ellipse is the geometrical plot of a system with internal fluctuations, and the decrease of AFE reflects the concentration of data and a greater stability of the system. In reverse, a larger AFE indicates a system irregularity or poor control of the physiological variable [24].

In this paper, we wanted to complete the experimental research towards an interval timing approach and the investigation of computational models of time estimation, based on the Poincaré plot method, which proved to be feasible for the study of RT variability [18]. We also wanted to determine the relationship between the linear conventional measure of RT and time estimation variability during serial testing (CV) and the nonlinear indexes of the Poincaré plot (SD_1_, SD_2_, SD_1_/SD_2_, and AFE), taking into account some categorical variables of the subjects, such as age, sex, health status, and anxiety level.

## 2. Materials and Methods

### 2.1. Study Design

We conducted a cross-sectional research on a sample of 180 subjects (50 men and 130 women, mean age 31.61 ± 13.56 years, minimum age 18 years, maximum age 80 years), selected from the Romanian population, based on voluntary acceptance to perform a computerized test. Each subject completed an online informed consent form for participants before the test, taking into account the ethical principles for research in human beings. The research was approved by the Ethics Committee of the Research Center for Promoting Excellence in Professional Training, University of Pitesti (reference number 1341/ 30 August 2021). Initially, a group of 75 students from the University of Pitesti was trained to apply the test. They each selected 3–5 acquaintances (family members, friends), who were tested according to the proposed procedure. We tried to cover a range as wide as possible in terms of the age of the subjects enrolled in the study. All participants had normal visual accuracy (with or without optical correction) and they did not report antecedents of important diseases (chronic or acute neuromotor pathology, somatosensory disorders, cognitive illness, etc.).

### 2.2. Data Acquisition

The study design was based on a web browser software program, developed for serial RT and time estimation testing. Thus, data gathering was based on a web platform, using the software PsyToolkit (https://www.psytoolkit.org/) (accessed on 4 November 2021) [25,26]. We used an online testing procedure for desktop computers or laptops, with two successive sections: a survey and the experiment, accessed by each participant through a specific link. The survey contained the informed consent form for participants and five items with closed ended questions for age, sex, self-reported health (SRH), self-reported anxiety (SRA), and laterality. The coded answers for each item were the following:

- Sex: male—1, female—2.
- Professional status: pupil/student—1, employee—2, unemployed—3, retired—4, household—5.
- SRH: excellent—1, very good—2, good—3, satisfactory—4, poor—5.
- SRA: not at all anxious—1, slightly anxious—2, moderately anxious—3, very anxious—4, extremely anxious—5.
- Laterality (hand used for writing): right—1, left—2.

**Figure 1:**
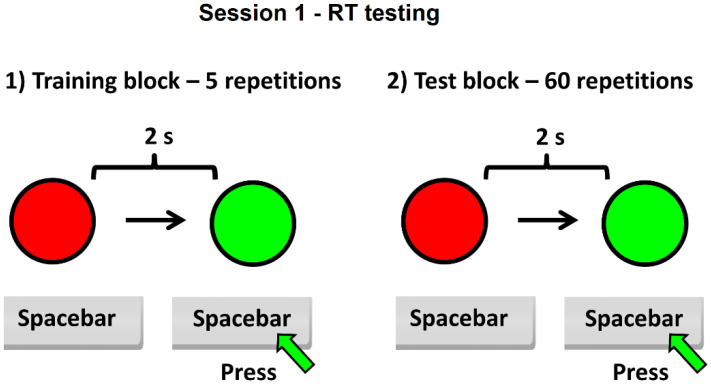
Schematic of the session 1 of the experiment—RT testing.

**Figure 2:**
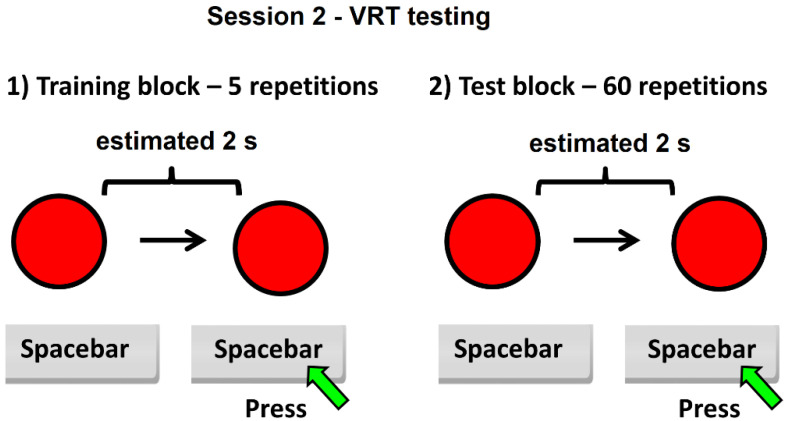
Schematic of session 2 of the experiment—VRT testing.

The experiment consisted of two sessions of testing: one for RT (Figure 1) and another for time estimation (called virtual reaction time—VRT) (Figure 2), each of them including a training block (with five repetitions, designed for the subject to become familiar with the test interface), followed by the test block itself (60 repeated tasks). The subject needs about 8–10 min to complete the test. For the first session (RT), the subject must press the space bar key very quickly, using the dominant hand, when the color of a circle from red to green is perceived on the screen. The time between two successive tasks was set to 2 s after pressing the spacebar key, the maximum allowed response time being 3 s. The program displays on the screen at the end of the first session the average value of the RT for 60 repetitive tasks.

For the VRT test, each subject must imagine that the color of the circle will change from red to green every 2 s. The subject must answer 60 times repeatedly, every estimated 2 s, by pressing the spacebar key. After pressing the spacebar key, for a fraction of a second, the color of the circle changes from red to green. The red color then reappears and a new interval starts, which must be estimated at 2 s. At the end of session 2, participants receive immediate feedback expressed as the average value for the estimated time intervals. For both sessions, the timing interval was set to 2 s to build up a stable reference memory of the standard duration of the task.

To remove outliers in the data set, for the RT test, subjects with any answer lasting more than 3 s were eliminated. Additionally, for the VRT test, we excluded from the analysis the subjects with any estimated interval larger than 7 s. Only the subjects who answered correctly and completed the experiment and who did not show a lack of attention or gave accidental responses were selected. To carry out the test, it was recommended to place the subject in an environment without sound or other disturbances, which would offer conditions of optimal concentration on the work task. Subjects were seated comfortably in a chair behind a table, facing a computer screen.

### 2.3. Statistical Analysis of Data

Data analysis was performed using the software IBM SPSS 20.0 (IBM Corp., Armonk, NY, USA) [27]. Thus, we calculated the mean, the standard deviation (SD), and the coefficient of variation (CV) of data, and we applied the Shapiro–Wilk test for data distribution and a test for reliability of the RT and VRT sessions of testing (the Cronbach’s alpha). We then realized a correlational analysis of data, based on parametric (the Pearson’s correlations) and non-parametric methods (Spearman’s rank correlation), followed by simple linear regression analysis.

We also calculated for VTR the Relative Reproduction Error (RRE) as the difference between the response (VRT) and the interval duration (2000 ms), normalized by the interval duration (2000 ms):

(1) $$\mathrm{RRE}=\frac{\left(\mathrm{VRT}-2000\right)}{2000}\times 100$$

This metric provides a measure of the degree of the estimation bias [11].

The statistical analysis was completed by applying the Poincaré plot method for the series of data, by determining the indicators that are needed: SD_1_, SD_2_, SD_1_/SD_2_, and AFE, according to the following formulas [21,22,28,29]:

(2) $${\mathrm{SD}}_{1}=\frac{\sqrt{2}}{2}*\mathrm{SD}\left(x_{n}-x_{n+1}\right)$$

(3) $${\mathrm{SD}}_{2}=\sqrt{2\mathrm{SD}{\left(x_{n}\right)}^{2}-\frac{1}{2}\mathrm{SD}{\left(x_{n}-x_{n+1}\right)}^{2}}$$

(4) $$\mathrm{AFE}={\pi *\mathrm{SD}}_{1}{*\mathrm{SD}}_{2}$$

In these formulas, SD(x_n_−x_n+1_) is the SD of the differences x_n_−x_n+1_ from the string of data, and, respectively, SD(x_n_) represents the SD of x_n_.

## 3. Results

### 3.1. Descriptive Statistics for the Study Group

The sampling bias was managed by using appropriate inclusion and exclusion criteria for the selection of participants [30]. Additionally, the involvement of subjects in testing tasks can be a possible source of bias response, caused by a variety of sequential effects observed in serial RT tasks [31], which can be managed by removing outliers [32]. In our case, the recruitment of the subjects was based on their assumed availability to carry out the testing procedure, and the recorded outliers determined the exclusion of the respective subjects.

From the initial number of study subjects (234) who accessed the experiment, we selected 180 valid participants (76.92%), according to the inclusion and exclusion criteria mentioned above. For the final study group, no outliers or missing values were reported. The main results of the data gathered are presented in Table 1, Table 2 and Table 3 as descriptive statistics (mean and standard deviation).

After checking the type of distribution of data by using the Shapiro–Wilk test, we determined that the data was not normally distributed. Overall, for the experimental group, the health status of the subjects (average score of SRH 2.32 ± 0.80) was between very good and good, while the anxiety level (average score of SRA 1.77 ± 0.79) showed no anxiety to slight anxiety.

### 3.2. Reliability of the RT and VRT Serial Tests

The internal consistency for the set of data obtained through each session of testing was excellent (Cronbach’s Alpha 0.97 for RT and 0.99 for VRT).

### 3.3. Correlation and Regression Analysis of Data

To measure the strength and direction of association existing between the recorded variables, we applied a parametric correlation analysis (Pearson’s correlation) for the ratio scale variables and a non-parametric correlation analysis (Spearman’s rank correlation) for ordinal variables (Table 4).

Given the statistically significant correlations and importance in terms of intensity obtained, we ran a regression analysis for linear models of relevant variables. This type of regression is suitable for data with normal and non-normal distributions if the group of subjects is large [33]. Even if our data did not have a normal distribution, the residuals (errors) of the regression line were approximately normally distributed. This assumption was checked through two methods: the histograms (with a superimposed normal curve) and the normal P-P Plots. The assumption of normally distributed errors is important to linear regression [34], but in large sample sizes (e.g., where the number of observations per variable is higher than 10), violations of this rule have only limited effect on results [35,36].

The regression models for age (independent variable) significantly predict the dependent variables RT and VRT (Table 5).

The same procedure was performed for the relation between CV of RT (independent variable) and the Poincaré plot descriptors (dependent variables) (Table 6), respectively, between CV of VRT (independent variable) and the Poincaré plot descriptors (dependent variables) (Table 7).

The results showed that the overall regressions were statistically significant. To estimate the effect size for the regression models, we analyzed the adjusted R square values. According to Cohen (1988), an R-square value ≤0.12 indicates a small effect size, between 0.13 to 0.25 indicates a medium effect size, and >0.26 indicates a large effect size [37]. Thus, we put into evidence a large effect size for the effect of the CV of RT on SD_1_, SD_2_, and AFE and for the effect of the CV of VRT on SD_2_ and AFE. Additionally, a medium effect size was reported for the effect of age on RT and for the effect of the CV of VRT on SD_1_. On the other hand, a small effect size was recorded for the effect of age on VRT, the effect of the CV of RT on SD_1_/SD_2_, and the effect of the CV of VRT on SD_1_/SD_2_.

## 4. Discussion

### 4.1. The Study Implications

The results indicated the excellent level of internal consistency of the RT and VRT serial testing as an indicator of the analogy of the repetitive responses for each session (Cronbach’s Alpha 0.991 for RT session and 0.99 for VRT session). We validated two instruments that could be effectively used for determining consistent response patterns in reaction time and time estimation research. Other authors have also shown that the process of time estimation represents a paradigm in terms of the reliability of the required tasks [38].

Our data gathered through the proposed experimental design offered the opportunity for a comparative approach of linear and nonlinear analysis. In addition, we included a new method for nonlinear dynamic analysis of parameters’ variability in the form of the method of the Poincaré plot. We have arguments to believe that this method was used for the first time in our research, fully demonstrating its usefulness. Thus, we performed statistical data processing using two types of recorded variables: categorical variables (age, sex, SRH, SRA) and psychometric variables (RT and VRT). For these data series, we measured indicators of central tendency and dispersion (mean, standard deviation, CV, and RRE), and we then applied a nonlinear method of analysis for RT and VRT variability, based on Poincaré plot descriptors (SD_1_, SD_2_, SD_1_/SD_2_, and AFE).

The Poincaré method has wide applications in the study of physiological and biomechanical parameters and assessment of autonomous control for data series that involve dependency between consecutive observations or not [28,39]. Thus, a recent study has proved the utility of the method to quantify physical activity variability by using Fitbit devices, with the following trackers: steps per day, distance per day, daily minutes of being lightly active, fairly active, and very active [40]. The authors took into consideration relatively independent parameters, analyzed in a series of data with nonlinear dynamics. In our study, we consider that serial testing of RT and VRT implied identical repetitive tasks, performed with the same resources and involving the same neurophysiological mechanisms. Therefore, the variability of the tested parameters could be successfully analyzed through the Poincaré indices, which reflect the oscillations of the functioning of a biological system during repetitive work tasks, conditioned by the intervention of neurocognitive processes. Our model of analysis was designed, starting from the applicability of the Poincaré plot in the study of heart rate and respiration patterns [22,41].

The matrix of correlations between the recorded variables (Table 4) put into evidence a medium correlation between the subjects’ age and RT (*p* < 0.05) and a small correlation between the subjects’ age and VRT (*p* < 0.05). From the output of linear regression analysis, 24% of the total variation in the dependent variable RT could be explained by the independent variable age (*p* < 0.001), while only 4% of the total variation in the dependent variable VRT could be explained by the independent variable age (*p* < 0.001). However, in interpreting these results, we must take into account the structure of the sample depending on age, 69.4% of subjects being under 40 years. As there was no homogeneity of the age groups in the studied sample, some precautions must be kept in generalizing the results in relation to the mentioned variables. From the same perspective of the characteristics of the subjects, selected according to the assumed criteria, mostly with excellent, very good, or good health (95.6% of subjects) and without anxiety or slight anxiety (83.3% of subjects), the analysis of the association between RT and VRT with SRH and SRA loses its relevance.

The relationship of RT with age has been much studied, starting from the idea that RT implies a neurophysiological mechanism of cognitive processing of information, which is obviously correlated with age [42]. It is well established that simple RT is influenced by age because the average values of RT and its variability grows with age, this being proven by comparisons between young people, adults, and older people [43]. Additionally, RT is a useful biomarker of physiological or pathological brain aging, neurogenesis, and neuroplasticity, which can be largely influenced by the interaction between social and health determinants [44]. On the other hand, the process of estimating time is also correlated with the aging process. In this case, the mechanisms involved are much more complex, the explanations offered by various authors varying very widely. In essence, it is about taking into account the existence of the internal clock, functionally correlated with the biological age of the individual [8]. Another perspective is provided by the attentional counter theory, which assumes that there is a cognitive timer that counts subjective time events [45]. Consequently, there is an age-related decline in temporal cognition that can explain complex neurophysiological processes associated with age-related dysfunctionalities of time estimation and time synchronization tasks, referring to intervals in the range of milliseconds to a few minutes [46,47].

Another aspect to note is the very small correlation between RT and VRT, respectively, between RT and RRE (r = −0.09). This result is in agreement with previous classical findings of other authors, according to whom there is apparent independence of simple RT and measured time-keeping ability [48]. In addition, simple motor RT and estimation of time intervals succession and duration are commonly recognized as two different methods for estimating perceptual latency [49]. However, beyond these differences, RT is widely used to understand how individuals perceive and discriminate different forms of time representations [50].

It should be noted that the average VRT in the experimental group was 1540.80.21 ± 592.63 ms, with an average RRE of −22.96 ± 29.63%, which indicates a global underestimation of the time interval of 2000 ms that was imposed for repetitive reproduction. The tendency to distort time intervals during their estimation is real and has been the subject of numerous research. Traditionally, Vierordt’s law considers that people tend to distort the length of reproduced time intervals of previous tasks, with lower estimates values in case of short intervals and higher estimates values in case of long intervals [51]. However, this applies when the experimental task imposes time intervals of minutes [51]. The phenomenon described above is known as the “central tendency” in time estimation [52]. In addition, the results of such tests depend on a multitude of factors, especially related to the test conditions [9]. Studies focused on time perception have also revealed inconsistent data when applying techniques to modify the excitability of the brain via transcranial stimulation. In these cases, the differences could be interpreted in the context of various experimental designs [53]. Thus, Koch et al., 2007, put into evidence an overestimation of the length of time intervals, while Jones et al., 2004, found temporal under-reproduction for time intervals ranging between 500 and 2000 ms [54,55].

The implicit or explicit nature of timing tasks also influences the way that time intervals are perceived. This difference refers to the fact that, during implicit time estimation requests, the subjects are not informed about the duration of the stimulus, while in the case of explicit variants, the subjects are required to respect a known duration of the stimulus [56]. The results of a relevant research revealed an overestimation of the implicit timing tasks for intervals of 500, 1000, and 2000 ms, while during explicit tasks, subjects tend to have larger estimations for time intervals of 500 ms and shorter estimations in case of intervals of 1000 and 2000 ms [57]. In our case, the repetitive task of estimating 2000 ms intervals in the form of a virtual reaction time was explicit. Therefore, in our case, the recorded underestimation of the time required for repetitive reproduction is actually in line with the above logic.

Next, the analysis of data was oriented towards the study of RT and VRT variability in terms of CV and Poincaré plot indexes. We started from the idea that the Poincaré plot method represents a complementary alternative to conventional tests for the study of the variability of data [58]. Taken together, the results of this study indicated that the mean CV of RT (33.04 ± 15.91%) was higher than the mean CV of VRT (15.21 ± 8.82%) for the same number of repetitive tasks (60).

The CV in time estimation research is considered to be equivalent to the WF, according to Scalar Timing Theory, and it is correlated with the process of neural coding in time perception [6,11]. Our mean value for the CV of VRT can be compared with the mean values of the WF reported by various studies. Usually, time estimation studies refer to the quantitative assessment of the length of time intervals during visual or auditory conditions of stimulation, a situation in which the WF is applied. Thus, for experiments of time estimation with filled auditory intervals, WFs were observed in the range of 10–13% for durations between 400 and 2000 ms [59]. In the case of time estimation research with visual stimuli, the WF varies between 12% and 16% for durations ranging from 150 to 900 ms [60]. Additionally, other authors have highlighted an inconsistency of the scalar property of time estimation when the time intervals are in the range of 1–2 s, especially in the conditions of variation of the number of reproduced intervals (from 1 to 5 intervals). From the mentioned research, an average value emerged of the WF of 10–12.5% for an estimated duration of 1900 ms [13]. The WF for one interval discrimination is smaller for a duration of 200 ms than for 2 s. Consequently, the interval of 2 s appears as a landmark for the cognitive processes of temporal information processing. [61,62]. In addition, the violation of Weber’s law is more obvious in the case of visual experimental tasks when compared with auditory ones [59].

In the present paper, each subject was asked to fractionate the flow of time into intervals of 2 s, based on visual stimuli, which is to estimate repeatedly short time intervals with a fixed duration of 2 s. Our results confirm the hypothesis of the scalar property of temporal representation by reporting the average value of CV of VRT to the existing nomograms. The considered reference data sets resulted from studies on the effects of interval length on time estimation precision in tests with repetitive tasks [63,64]. The repetitive and rhythmic character of such a testing design requires a flexible use of attentive functions in subjective time perception to reduce the process of time distortions [64].

Returning to our nonlinear analysis, the average Poincaré plot parameters for RT and VRT showed that session 1 of the experiment produced data with a lower dynamic variability than session 2. Linear regression analysis of Poincaré plot descriptors against conventional metric of RT and VRT variability (CV) indicated that SD_1_, SD_2_, and AFE had a consistently closer relationship with the CV of RT and with the CV of VRT (*p* < 0.01). The best regression models were for the relationship between the CV of RT and SD_1_, which explains 62% of the variability in SD_1_ (Table 5), and for the relationship between the CV of VRT and SD_2_, which explains 56% of the variability in SD_2_ (Table 6).

Other authors have also demonstrated the close relationship between CV and dynamic indices of the Poincaré plot in the case of other physiological variables, such as blood pressure [65,66] or blood sugar levels [58]. Since there is an interest for the simultaneous consideration of the mentioned parameters, as a novelty element, we proposed the application of this comparative analysis in the case of RT and VRT variability to display nonlinear aspects of the time-interval sequential patterns.

The CV represents a standardization of the standard deviation that allows comparison of variability estimates [67], and the Poincaré index reflects an estimation of the variability of a time-series as temporal aspects to the non-linear analysis [19]. The linear regression between CV and the Poincaré not only tested for relationships between variables but also quantified their direction and strength through the obtained regression coefficients [68]. Traditionally, CV is used in the studies of the variability of RT [69] and time estimation, when it is superimposable with WF values [11]. Our regression models have shown that we can extend the analysis of RT and VRT variability from the classic CV determination model by calculating the Poincaré indices. The new method, applicable to various physiological time-series of data to reveal the adaptation of a system that deals with environmental, physiological, and sometimes pathological factors, is also relevant in the case of the study of RT and VRT variability [19].

In conclusion, both CV and Poincaré plot descriptors are useful for a complementary assessment of RT and VRT variability because the initial linear analysis of the data series can then be completed by a nonlinear analysis. The demonstrated relationship between CV and Poincaré plot indexes in the case of VRT allows, in addition, the description of the nonlinear dynamics of the time estimation process and to put into evidence hidden correlation patterns for a time series of data. This fact gives additional arguments to the idea that the subjective representation of time is a nonlinear mapping, with stochastic character, and is possibly determined by the nonlinear neural representation of time intervals [13,70].

### 4.2. Limitations of the Study

Our study had some limitations, especially in terms of subject selection. Thus, we did not use a randomization methodology in assigning subjects, but we found subjects based on their willingness to participate in the study. Additionally, there are some methodological limitations regarding the data collecting, such as the relevance of subjects’ answers for the items of the questionnaire and their involvement and performance in the execution of the required tasks. We also point out that the experimental design was based on the go RT procedure (the subject press a button when one visual stimulus appears) and not the stop RT procedure. Thus, the stop RT task measures inhibition of a response that has already been initiated [71] and has proven to be an important indicator of the cognitive control processes that are involved in stopping tasks [72]. The go RT is the classical response to the stimulus presented on the computer screen [73]. Some authors claim the existence of a common mechanism for inhibiting and switching reactions in both types of tasks, but it seems that experiments with frequent go RT tasks determine greater response bias [74].

### 4.3. Future Research Directions

Our results can lead to new lines of research in the area of RT and VRT. Thus, the mathematical modelling of data using the Poincaré method, and the facile use of the Web testing platform, offer opportunities to expand research on larger samples of subjects, across different age groups. Additionally, such research can be performed in the case of subjects with different morbidities or occupational contexts, taking into account composite variables.

## 5. Conclusions

The present study provides novel evidence that linear and nonlinear analysis of RT and VRT variability during serial testing bring complementary insights to the understanding of complex neurocognitive processes implied in the task execution. The mathematical modeling of data allowed to put into evidence a similar significant relationship of association of the RT and VRT with the age of the subjects, which was more pregnant for RT (*p* < 0.05). Our obtained results also showed that the dynamic of RT and VRT determined a consistent closer association between CV and the Poincaré plot descriptors SD_1_, SD_2_, and AFE (*p* < 0.01). Finally, our findings suggested an underestimation of the time interval of 2 s that was imposed for repetitive reproduction for the VRT session of testing. The average value of CV of VRT (15.21 ± 8.82%) during the repetitive short-time estimation test, with visual stimuli, as an equivalent of the WF, confirms the hypothesis of the scalar property of temporal representation.

## Figures and Tables

**Table 1:** Descriptive parameters of the experimental group, based on the questionnaire answers (*n* = 180).

Variable	Age Years	SRH	SRA
Mean	31.61	2.32	1.77
SD	13.56	0.80	0.79

Abbreviations: SRH, self-reported health; SRA, self-reported anxiety; SD, standard deviation; *n*, group size.

**Table 2:** Descriptive parameters of the experimental group, based on the RT session of testing (*n* = 180).

Variable	RT ms	CV %	SD_1_ ms	SD_2_ ms	AFE ms^2^	SD_1_/SD_2_
Mean	263.94	33.04	80.60	87.76	28,070.77	0.93
SD	69.17	15.91	42.90	47.59	36,014.82	0.16

Abbreviations: RT, reaction time; CV, coefficient of variation; SD, standard deviation; SD_1_, SD_2_, AFE, SD_1_/SD_2_, Poincaré plot descriptors; *n*, group size.

**Table 3:** Descriptive parameters of the experimental group, based on the VRT session of testing (*n* = 180).

Variable	VRT ms	CV %	RRE %	SD_1_ ms	SD_2_ ms	AFE ms^2^	SD_1_/SD_2_
Mean	1540.80	15.21	−22.96	170.88	265.34	191,331.54	0.69
SD	592.63	8.82	29.63	109.59	189.00	305,745.98	0.24

Abbreviations: VRT, virtual reaction time; CV, coefficient of variation; RRE, Relative Reproduction Error; SD, standard deviation; SD_1_, SD_2_, AFE, SD_1_/SD_2_, Poincaré plot descriptors; *n*, group size.

**Table 4:** Correlation output and level of statistical significance *p* (*n* = 180).

Variable	Age	Sex	SRH	SRA	RT	CV RT	SD_1_ RT	SD_2_ RT	AFE RT	SD_1_/SD_2_ RT	VRT	CV VRT	RRE	SD_1_ VRT	SD_2_ VRT	AFE VRT	SD_1_/SD_2_ VRT
**Age**	1.00 ^b^																
**Sex**	−0.12 ^a^	1.00 ^a^															
**SRH**	0.15 ^a^*	0.14 ^a^	1.00 ^a^														
**SRA**	−0.12 ^a^	0.11 ^a^	0.29 ^a^*	1.00 ^a^													
**RT**	0.49 ^b^*	0.13 ^a^	0.12 ^a^	−0.04 ^a^	1.00 ^b^												
**CV RT**	−0.15 ^b^*	−0.05 ^a^	−0.06 ^a^	0.14 ^a^	−0.24 ^b^*	1.00 ^b^											
**SD_1_ RT**	0.10 ^b^	0.03 ^a^	0.00 ^a^	0.14 ^a^	0.32 ^b^*	0.79 ^b^*	1.00 ^b^										
**SD_2_ RT**	0.15 ^b^*	0.03 ^a^	−0.03 ^a^	0.10 ^a^	0.46 ^b^*	0.71 ^b^*	0.92 ^b^*	1.00 ^b^									
**AFE RT**	0.19 ^b^*	0.03 ^a^	−0.01 ^a^	0.12 ^a^	0.51 ^b^*	0.61 ^b^*	0.92 ^b^*	0.93 ^b^*	1.00 ^b^								
**SD_1_/SD_2_ RT**	−0.17 ^b^*	0.06 ^a^	0.02 ^a^	0.16 ^a^*	−0.31 ^b^*	0.24 ^b^*	0.24 ^b^*	−0.10 ^b^	0.02 ^b^	1.00 ^b^							
**VRT**	−0.21 ^b^*	−0.01 ^a^	0.03 ^a^	0.07 ^a^	−0.09 ^b^	0.04 ^b^	−0.01 ^b^	−0.03 ^b^	−0.06 ^b^	0.01 ^b^	1.00 ^b^						
**CV VRT**	−0.03 ^b^	−0.15 ^a^*	−0.04 ^a^	0.03 ^a^	0.17 ^b^*	0.00 ^b^	0.14 ^b^	0.13 ^b^	0.16 ^b^*	0.06 ^b^	−0.12 ^b^	1.00 ^b^					
**RRE**	−0.21 ^b^*	−0.01 ^a^	0.03 ^a^	0.07 ^a^	−0.09 ^b^	0.04 ^b^	−0.01 ^b^	−0.03 ^b^	−0.06 ^b^	0.01 ^b^	1.00 ^b^	−0.12 ^b^	1.00 ^b^				
**SD_1_ VRT**	−0.15 ^b^*	0.11 ^a^	0.04 ^a^	0.06 ^a^	0.12 ^b^	−0.01 ^b^	0.10 ^b^	0.10 ^b^	0.09 ^b^	0.04 ^b^	0.57 ^b^*	0.49 ^b^*	0.57 ^b^*	1.00 ^b^			
**SD_2_ VRT**	−0.14 ^b^	0.02 ^a^	−0.02 ^a^	0.03 ^a^	0.11 ^b^	0.03 ^b^	0.12 ^b^	0.12 ^b^	0.11 ^b^	0.02 ^b^	0.49 ^b^*	0.75 ^b^*	0.49 ^b^*	0.76 ^b^*	1.00 ^b^		
**AFE VRT**	−0.14 ^b^	0.05 ^a^	0.02 ^a^	0.06 ^a^	0.11 ^b^	0.03 ^b^	0.11 ^b^	0.14 ^b^	0.11 ^b^	−0.03 ^b^	0.46 ^b^*	0.61 ^b^*	0.46 ^b^*	0.90 ^b^*	0.87 ^b^*	1.00 ^b^	
**SD_1_/SD_2_ VRT**	0.06 ^b^	0.08 ^a^	0.15 ^a^*	0.06 ^a^	−0.01 ^b^	−0.08 ^b^	−0.07 ^b^	−0.09	−0.08 ^b^	0.05 ^b^	0.02 ^b^	−0.27 ^b^*	0.02 ^b^*	0.16 ^b^*	−0.35 ^b^*	−0.07 ^b^	1.00 ^b^

Abbreviations: SRH, self-reported health; SRA, self-reported anxiety; RT, reaction time; VRT, virtual reaction time; CV, coefficient of variation; RRE, relative reproduction error; SD_1_, SD_2_, AFE, SD_1_/SD_2_, Poincaré plot descriptors; ^a^, Spearman’s rank correlation coefficient; ^b^, Pearson’s correlation coefficient; *, *p* < 0.05 was considered statistically significant (2-tailed); *n*, group size.

**Table 5:** Model summary, ANOVA report and coefficients for simple linear regression analysis—age versus RT and VRT (*n* = 180).

Variable	R	R Square	Adjusted R Square	SE	F	*p*	β0	SE	*p*	95%LB	95%UB	β1	SE	*p*	95%LB	95%UB
**RT**	0.49	0.24	0.23	60.65	54.84	0.001	185.67	11.49	0.001	162.99	208.36	2.48	0.33	0.001	1.82	3.14
**VRT**	0.21	0.04	0.04	581.16	8.13	0.005	1829.62	110.15	0.001	1612.25	2046.98	−9.14	3.20	0.005	−15.46	−2.81

Abbreviations: RT, reaction time; VRT, virtual reaction time; R, Pearson’s coefficient of correlation; SE, standard error; F, test for overall significance for the linear model; *p*, level of statistical significance; β0, the intercept coefficient; β1, the regression coefficient; 95%LB and 95%UB, lower bound and upper bound of the 95% confidence interval; *n*, group size.

**Table 6:** Model summary, ANOVA report and coefficients for simple linear regression analysis—CV of RT versus Poincaré plot descriptors (*n* = 180).

Variable	R	R Square	Adjusted R Square	SE	F	*p*	β0	SE	*p*	95%LB	95%UB	β1	SE	*p*	95%LB	95%UB
**SD_1_**	0.79	0.62	0.62	26.45	292.91	0.001	10.32	4.55	0.025	1.34	19.32	2.13	0.12	0.001	1.88	2.37
**SD_2_**	0.71	0.51	0.50	33.55	182.18	0.001	17.48	5.78	0.003	6.08	28.88	2.13	0.16	0.001	1.82	2.44
**AFE**	0.61	0.37	0.36	28,713.49	103.61	0.001	−17,295.44	4944.17	0.001	−27,052.2	−7538.71	1373.2	134.9	0.001	1106.97	1639.42
**SD_1_/SD_2_**	0.24	0.06	0.05	0.16	10.78	0.001	0.85	0.03	0.001	0.79	0.9	0.002	0.001	0.001	0.001	0.004

Abbreviations: RT, reaction time; R, Pearson’s coefficient of correlation; SE, standard error; F, test for overall significance for the linear model; *p*, level of statistical significance; β0, the intercept coefficient; β1, the regression coefficient; 95%LB and 95%UB, lower bound and upper bound of the 95% confidence interval; SD_1_, SD_2_, AFE, SD_1_/SD_2_, Poincaré plot descriptors; *n*, group size.

**Table 7:** Model summary, ANOVA report and coefficients for simple linear regression analysis—CV of VRT versus Poincaré plot descriptors (*n* = 180).

Variable	R	R Square	Adjusted R Square	SE	F	*p*	β0	SE	*p*	95%LB	95%UB	β1	SE	*p*	95%LB	95%UB
**SD_1_**	0.49	0.24	0.24	95.60	57.23	0.001	77.72	14.23	0.001	49.64	105.8	6.13	0.81	0.001	4.53	7.73
**SD_2_**	0.75	0.56	0.56	125.47	228.15	0.001	21.21	18.67	0.001	−15.64	58.06	16.06	1.06	0.001	13.96	18.15
**AFE**	0.61	0.37	0.36	243,925.9	103.23	0.001	−127,906	36,301.7	0.001	−199,543.1	−56,268.9	20,995	2066	0.001	16,917	25,072.7
**SD_1_/SD_2_**	0.27	0.07	0.07	0.23	14.08	0.001	0.8	0.03	0.001	0.74	0.87	−0.007	0.002	0.001	−0.011	−0.003

Abbreviations: VRT, virtual reaction time; R, Pearson’s coefficient of correlation; SE, standard error; F, test for overall significance for the linear model; *p*, level of statistical significance; β0, the intercept coefficient; β1, the regression coefficient; 95%LB and 95%UB, lower bound and upper bound of the 95% confidence interval; SD_1_, SD_2_, AFE, SD_1_/SD_2_, Poincaré plot descriptors; *n*, group size.

## Data Availability

The data are available on request from the corresponding author. All data relevant to the study are included in the article.

## Abbreviations

CV	the coefficient of variation
RT	reaction time
VRT	virtual reaction time
SD	standard deviation
AFE	area of the fitting ellipse
WB	Weber fraction
SRH	self-reported health
SRA	self-reported anxiety
RRE	Relative Reproduction Error
R	Pearson’s coefficient of correlation
SE	standard error
F	test for overall significance for the linear model
*p*	level of statistical significance
β0	the intercept coefficient
β1	the regression coefficient
95%LB	lower bound of the 95% confidence interval
95%UB	upper bound of the 95% confidence interval
*n*	group size
